# Combination of syringaresinol–di–O–β-d-glucoside and chlorogenic acid shows behavioral pharmacological anxiolytic activity and activation of hippocampal BDNF–TrkB signaling

**DOI:** 10.1038/s41598-020-74866-4

**Published:** 2020-10-23

**Authors:** Shouhei Miyazaki, Yoshio Fujita, Hirotaka Oikawa, Hideo Takekoshi, Hideaki Soya, Masato Ogata, Takahiko Fujikawa

**Affiliations:** 1 Laboratory of Molecular Prophylaxis and Pharmacology, Graduate School of Pharmaceutical Sciences, Suzuka University of Medical Science, 3500-3 Minamitamagaki-cho, Mie, 513-8670 Japan; 2 Faculty of Pharmaceutical Sciences, Suzuka University of Medical Science, 3500-3 Minamitamagaki-cho, Mie, 513-8670 Japan; 3 Production and Development Department, Sun Chlorella Corp., 369 Osaka-cho, Karasuma-dori Gojo-sagaru, Shimogyo-ku, Kyoto, 600-8177 Japan; 4 Laboratory of Exercise Biochemistry and Neuroendocrinology, Faculty of Health and Sport Sciences, University of Tsukuba, Tsukuba, Ibaraki 305-8574 Japan; 5 Sport Neuroscience Division, Advanced Research Initiative for Human High Performance (ARIHHP), University of Tsukuba, Tsukuba, Ibaraki 305-8574 Japan; 6 Department of Biochemistry and Proteomics, Mie University Graduate School of Medicine, 2-174 Edobashi, Tsu, Mie 514-8507 Japan

**Keywords:** Neuroscience, Medical research, Preclinical research, Health care, Quality of life

## Abstract

Mental stress, such as anxiety and conflict, causes physiological changes such as dysregulation of autonomic nervous activity, depression, and gastric ulcers. It also induces glucocorticoid production and changes in hippocampal brain-derived neurotrophic factor (BDNF) levels. We previously reported that *Acanthopanax senticosus* HARMS (ASH) exhibited anxiolytic activity. Thus, we attempted to identify the anxiolytic constituents of ASH and investigated its influence on hippocampal BDNF protein expression in male Sprague Dawley rats administered chlorogenic acid (CHA), ( +)-syringaresinol–di–O–β-d-glucoside (SYG), or a mixture of both (Mix) for 1 week using the open field test (OFT) and improved elevated beam walking (IEBW) test. As with ASH and the benzodiazepine anxiolytic cloxazolam (CLO), Mix treatment significantly increased locomotor activity in the OFT. CHA and Mix increased the time spent in the open arm in the IEBW test. SYG and Mix treatment inhibited the significant increase in normalized low-frequency power, indicative of sympathetic nervous activity, and significant decrease in normalized high-frequency power, indicative of parasympathetic nervous activity, as observed in the IEBW test. SYG and Mix treatment significantly increased hippocampal BDNF protein expression. The combination of CHA and SYG possibly induces anxiolytic behavior and modulates autonomic regulation, activates hippocampal BDNF signaling as with ASH.

## Introduction

Stress, induced by alterations of the external environment, affects the autonomic nervous system (ANS) as well as the hypothalamus–pituitary–adrenal (HPA) axis. ANS is largely divided into two branches: sympathetic (SNS) and parasympathetic nervous systems (PNS). SNS activation induces heart rate and blood pressure elevation, blood flow shift from the skin and viscera to the skeletal muscles, tracheal and bronchial smooth muscle relaxation. In contrast, PNS activation induces reverse effects. Anxiety (psychological stress) induces SNS activation and represses PNS^1^. This phenomenon has been demonstrated in patients with anxiety disorder and rodents under psychological stress^2–4^. The HPA axis induces various responses to counter environmental change (partially overlapping with SNS) by stimulating the secretion of steroid hormones and adrenaline into circulation. Chronic stress, such as long-term anxiety, induces gastric ulcers and depression via activation of the HPA axis and ANS^5,6^. Methods for relieving stresses are important for preventing stress-related diseases. Supplementation is a convenient method for people who are unable to manage stress. Among such supplements, plants have attracted attention globally^7,8^.

*Acanthopanax senticosus* HARMS (ASH) is a deciduous shrub and a member of the Araliaceae family that grows abundantly in Russia, China, and northern Japan. In China, ASH root bark is traditionally used to treat hypertension, mental disorders, and rheumatoid arthralgia^9^. In western countries, ASH is widely used in alternative medicine. ASH is also known as an adaptogen that increases resistance to stress^10,11^. ASH exhibits protective effect against gastric ulcers^12^ and prevents depression symptoms^13–15^. ASH extract exerts anxiolytic effects and modulates ANS activity in novelty-suppressed feeding and improves elevated beam walking (IEBW) test results^16^. ASH is suitable for daily supplementation based on its limited side effects^17,18^.

Recent studies identified the bioactive components of ASH, including syringing, chlorogenic acid (CHA), ( +)-syringaresinol di–O–β-D-glucoside (SYG, also called eleutheroside E), and isofraxidin. ASH extracts have also been characterized for pharmacological activity^9,19^. ASH prolongs swimming time, and SYG, but not CHA, has similar effects^12,20^. We previously reported that ASH extract prevents the onset of gastric ulcers induced by restraint stress in water^12^. CHA and SYG, which are present in high concentrations among ASH extract components, also prevent stress-induced gastric ulcer^12^. SYG was reported to have anti-inflammatory effects^21,22^, protective effects against cerebral ischemia^23,24^, and beneficial effects on insulin resistance^25^. In addition, SYG improves behavioral impairment induced by sleep deprivation^26^. Syringaresinol, the aglycone of SYG, also displays neuromodulating effects on presynaptic transmitter release^27^. CHA is well-known as an antioxidant^28^ and may show various positive effects on health^29^. The anxiolytic effects of CHA induced via GABA_A_ receptors has been reported^30^. These findings suggest that psychological stress resistance induced by ASH occurs through effects on the central nervous system (CNS). However, the contributions of individual components of ASH extract on anxiolytic effects are unknown.

Brain-derived neurotrophic factor (BDNF) plays an important role in neurogenesis, synaptic plasticity, and cognition and mood^31–34^. Recent studies report that increased BDNF expression in the hippocampus is associated with anxiolytic effects^35–40^. Stress-induced glucocorticoids reduce BDNF expression and impair synaptic plasticity and memory in the hippocampus^41–43^. ASH has protective effects against neurotoxicity^44–47^ and increases BDNF mRNA levels^48^. We previously reported that ASH increases hippocampal BDNF protein levels^16^. Therefore, the anxiolytic/anti-depressive effects of ASH may occur through the regulation of BDNF expression and its downstream signaling. However, components of ASH that contribute to effects on hippocampal BDNF signaling in the hippocampus have not been investigated.

In the present study, we examined the behavioral and anxiolytic effects of SYG, CHA, and their mixture (Mix) using open field (OFT) and IEBW tests. The latter is an improved version of the elevated plus maze (EPM) test. In the IEBW test, we assessed ANS activity via electrocardiography (ECG). We simultaneously performed western blotting and immunohistochemical analyses to explore possible mechanisms involving BDNF signaling that may be fundamental in the anti-anxiety effects of SYG, CHA, and Mix.

## Results

### OFT

**Figure 1:**
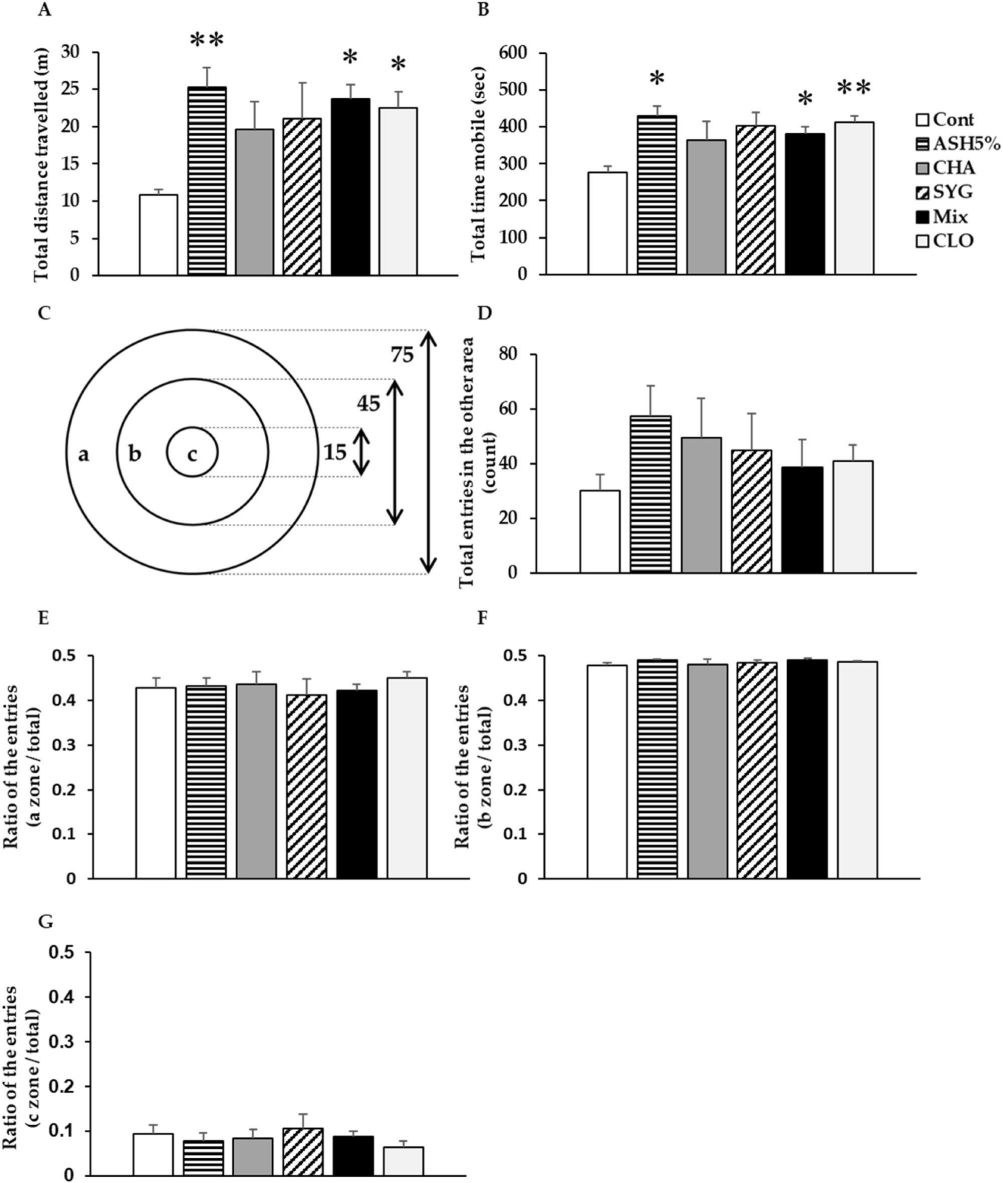
Effects of *Acanthopanax senticosus* HARMS (ASH), its major components (chlorogenic acid [CHA], ( +)-syringaresinol di–O–β-D-glucoside [SYG], a mixture of both [MIX]), and cloxazolam (CLO) on anxiety-related behaviors induced by mild stress in the open field test. (**A**) Total distance traveled. (**B**) Total time mobile. (**C**) The zone sectioning of the open field apparatus. The diameter of the apparatus was written in centimeters. (**D**) Total entries into another area (count). (**E**) The ratio of entries into another zone to the total entries. (**F**) The ratio of entries into zone b to the total entries. (**G**) The ratio of entries into zone c to the total entries. Data are presented as means ± SE; n = 5–6; *p < 0.05, **p < 0.01 versus control (Cont) (Dunnett’s *t*-test).

The total distance traveled and the total time mobile were significantly higher in the ASH 5%, Mix, and cloxazolam (CLO, benzodiazepine anxiolytic) rats than that in control (Cont) animals (Fig. 1A,B). CHA and SYG also increased these parameters, but differences were not significantly different from controls. We divided our apparatus into three sections, as presented in Fig. 1C, and measured the number of entries into each area. Total entries to another area tended to increase by various degrees in treated rats (Fig. 1D). We found no differences in the ratio of numbers of entries into each area to the total number of entries among the groups (Fig. 1E–G).

### IEBW test

The EPM test was used to evaluate animal anxiety. We attempted the assessment of ANS activity by heart rate variability (HRV) analysis. However, variability of the rat autonomic nervous activity in the EPM was large. We then used the IEBW apparatus by installing a 2 × 4 timber (180 × 8.9 cm) 190 cm above the floor level. The apparatus included open (140 × 8.9 cm) and closed (40 × 8.9 × 28.5 cm) arms (see  reference^16^ and supplementary materials for details). Rats were placed on the tip of the open arm, then allowed to explore the area freely for 3 min. We examined only those rats in which the ANS was not disturbed by the handling for reducing the variability.

#### Behavior in the IEBW test

**Figure 2:**
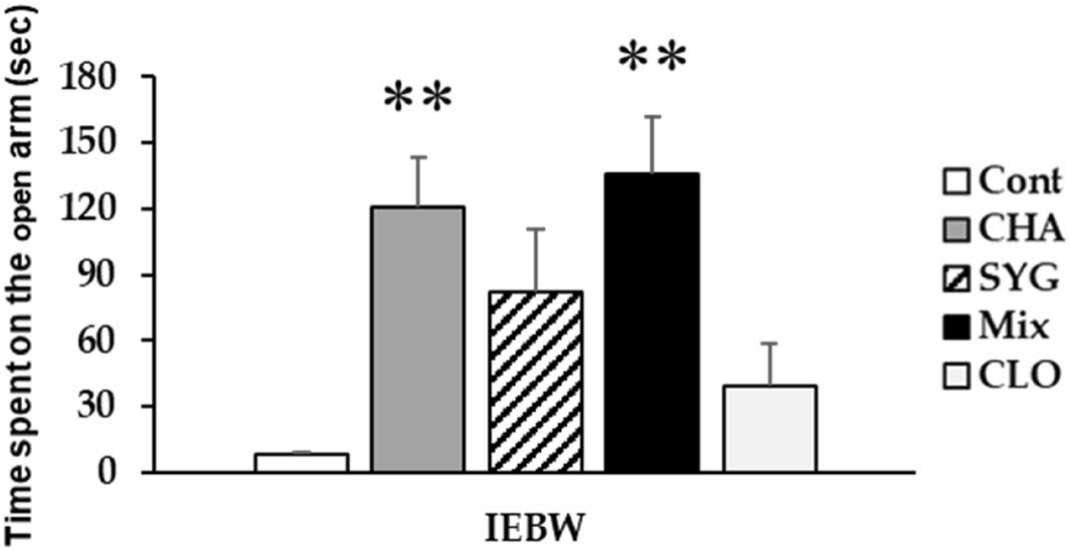
Effects of the major components of *Acanthopanax senticosus* HARMS (chlorogenic acid [CHA], ( +)-syringaresinol di–O–β-D-glucoside [SYG], and a mixture of both [MIX]) and cloxazolam (CLO) on anxious behaviors induced by substantial stress in the improved elevated beam walking test. Data are presented as means ± SE; n = 5–7; **P < 0.01 vs the control (Cont) group (Dunnett’s T3 test).

In this study, time spent on the open arm of the IEBW test was significantly increased in the CHA and Mix groups compared with the Cont group (Fig. 2). SYG-administered rats also exhibited a longer time spent on the open arm, but the difference was not significant from control animals. Staying time in rats treated with CLO, used as a positive control, was shorter than that in the SYG-treated rats. Upon return to the closed arm in the CLO group, remarkable muscle relaxation was observed. Further, the number of rats that slept was also increased in this group (data not shown).

#### ANS activity [heart rate variability (HRV) analysis]

**Figure 3:**
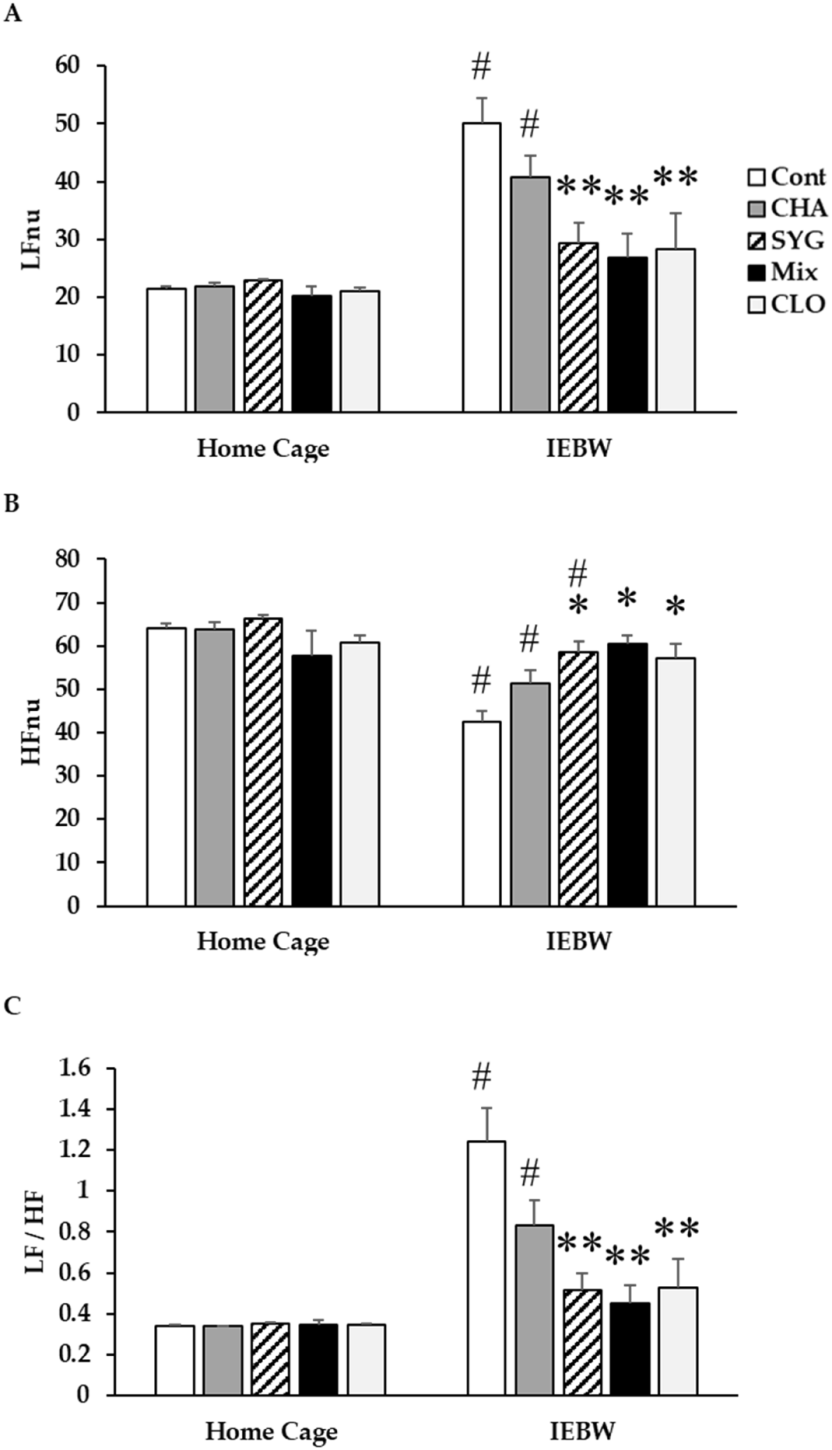
Effects of the major components of *Acanthopanax senticosus* HARMS (chlorogenic acid [CHA], ( +)-syringaresinol di–O–β-D-glucoside [SYG], and a mixture of both [MIX]) and cloxazolam (CLO) on autonomic nervous system control of heart rate in the improved elevated beam walking (IEBW) test. Power spectrum analysis of heart rate variability in the home cage and in the IEBW test. (**A**) Normalized low-frequency (LF) power (LFnu) reflects sympathetic nervous system activity. (**B**) Normalized high-frequency (HF) power (HFnu) reflects parasympathetic nervous system activity. (**C**) LF/HF indicates the balance between sympathetic and parasympathetic nervous system activities. Data are presented as means ± SE; n = 5–8; *p < 0.05, **p < 0.01 vs. the control (Cont) group (Dunnett’s *t*-test), ^#^*p* < 0.05 versus each home cage (paired *t*-test).

We placed rats implanted with a telemeter under home cage housing conditions and measured ANS activity at rest. Home cage housing did not alter ANS activity or low-frequency (LF)/high-frequency (HF) values in any group (Fig. 3). We subsequently measured ANS activity under IEBW conditions. IEBW conditions significantly increased normalized LF (LFnu) values and decreased normalized HF (HFnu) values in the Cont group compared with the findings under home cage conditions (Fig. 3A,B). In addition, we also observed significantly increased LF/HF ratios (Fig. 3C). Under IEBW conditions, the CHA group exhibited significantly higher LFnu and lower HFnu values than the findings under home cage conditions. Similarly, HFnu values were significantly decreased in the SYG group compared with those under home cage conditions. The administration of Mix and CLO under IEBW conditions suppressed changes in LFnu and HFnu values observed in the Cont group. Hence, compared with control animals under IEBW conditions, SYG, Mix, and CLO treated animals displayed significantly increased HFnu values and decreased LFnu and LF/HF values (Fig. 3A–C). However, the changes were not significant in the CHA treated rats. When considering behavioral results, the effects of CLO may be the result of muscle relaxation or sleep in comparison with those of CHA and Mix.

### Western blotting

**Figure 4:**
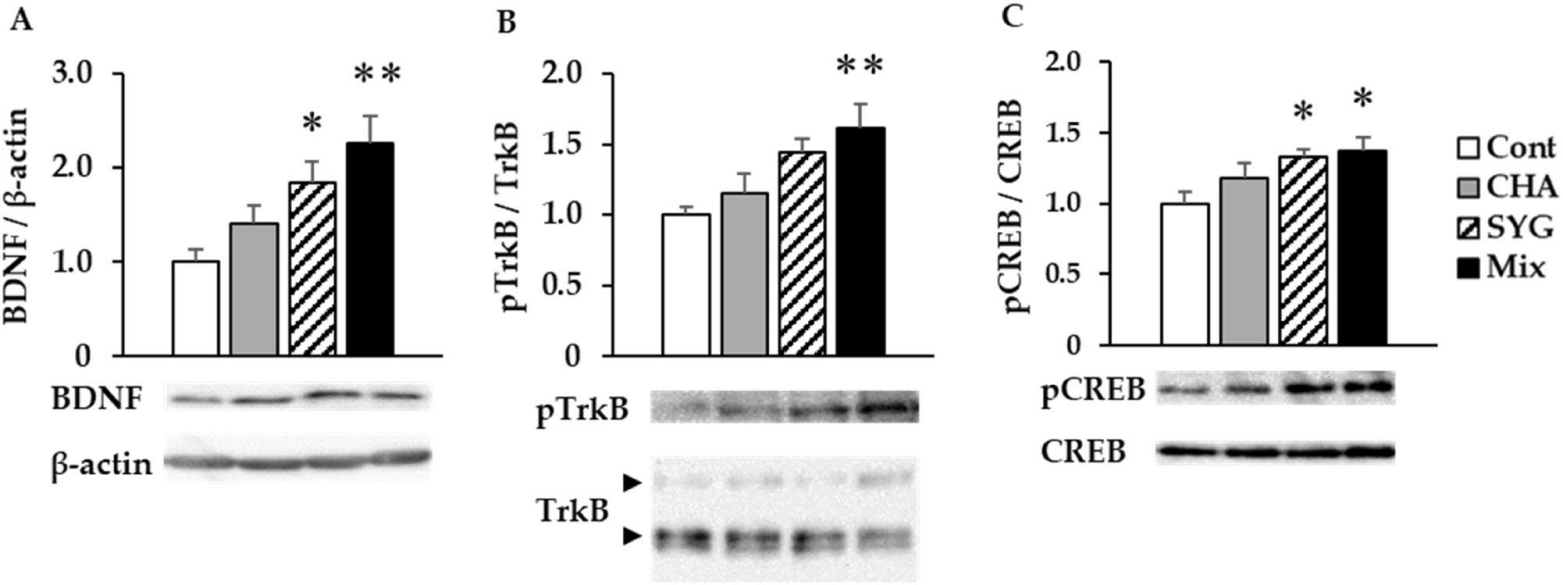
Effects of the major components of *Acanthopanax senticosus* HARMS (chlorogenic acid [CHA], ( +)-syringaresinol di–O–β-D-glucoside [SYG], and a mixture of both [MIX]) on hippocampal brain-derived neurotrophic factor (BDNF) signaling. Analysis of hippocampal (**A**) BDNF, (**B**) phospho-tropomyosin receptor kinase B (pTrkB), and (**C**) phospho-cAMP response element-binding protein (pCREB) protein levels by western blotting. BDNF expression was normalized to β-actin expression, whereas pTrkB and pCREB levels were normalized to those of TrkB and CREB, respectively. Each value is presented as a ratio vs the control (Cont) group. Data are presented as means ± SE; n = 5; *p < 0.05, **p < 0.01 vs Cont group (Dunnett’s *t*-test). Full-length blots are presented in Supplementary Fig. 1.

In the Cont, CHA, SYG, and Mix group rats, we resected the hippocampus from the brain and detected BDNF signaling-related protein expression and phosphorylation via western blotting. The hippocampal BDNF protein expression was significantly increased in the SYG and Mix groups but not in CHA animals (Fig. 4A). Moreover, in the Mix group, phosphorylation of tropomyosin receptor kinase B (TrkB), a BDNF receptor, was significantly elevated, followed by a significant increase in the phosphorylation of cAMP response element-binding protein (CREB), which is related to the transcriptional enhancement of BDNF mRNA (Fig. 4B,C). Further, CREB phosphorylation was significantly increased in the SYG group, but a clear increase of TrkB phosphorylation was not observed, in line with the findings in Mix group rats (*p* = 0.055).

### Immunohistochemistry

**Figure 5:**
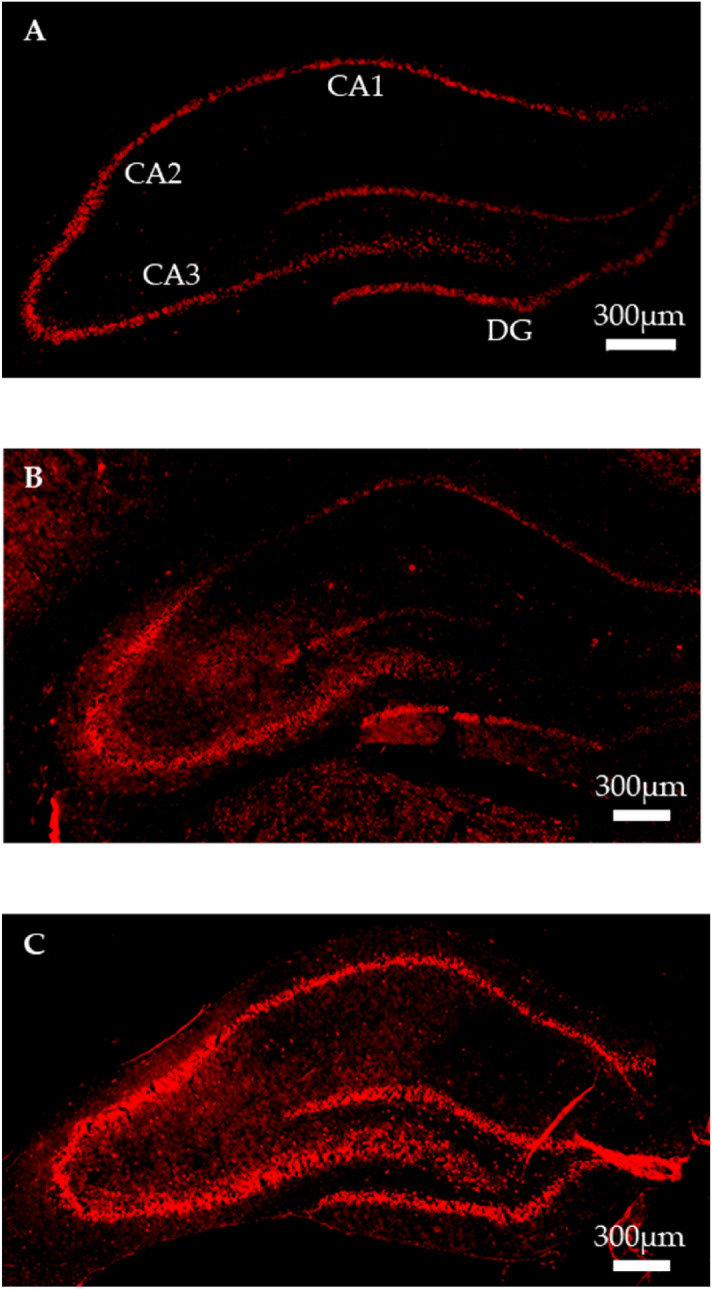
Immunohistochemical staining for hippocampal brain-derived neurotrophic factor (BDNF). Representative images of staining using anti-BDNF antibody in the (**A**) control (Cont), (**B**) ( +)-syringaresinol di–O–β-D-glucoside (SYG), and (**C**) mixture of SYG and chlorogenic acid (Mix) groups. CA1–3, cornu ammonis 1–3; DG, dentate gyrus.

We examined immunohistological changes in hippocampal BDNF protein expression in the SYG and Mix group rats. We observed a marked increase in BDNF protein expression in the rat hippocampus in both groups, in line with the western blot findings (Fig. 5). It was notable that the increase of BDNF expression in SYG only occurred in some regions, such as cornu ammonis [CA] 2–3 and dentate gyrus [DG] (Fig. 5B). In contrast, Mix group animals showed increased expression of BDNF across the entire CA and DG regions (Fig. 5C).

## Discussion

Using the OFT, Jin L. et al.reported that ASH extract does not change numbers of zone crosses and rearing in mice^14^. Thus, we investigated the number of entries into the center area as well as horizontal activity and mobile time in rats using the OFT. Locomotor activity in a novel environment was significantly higher in the ASH, Mix, and CLO group than that in the Cont group rats. However, the number of entries into the center area, which is a key anxiety-like behavior, was not significantly changed in these groups. ASH and Mix also ameliorated behavioral restraint induced by anxiety or fear in novel environments; however, they did not affect thigmotaxis, as reported by Jin L. et al*.* The increase in locomotor activity in the open field due to benzodiazepines is believed to be caused by the cancellation of anxiety-induced behavioral inhibition (disinhibition). Thus, the increase in activity seen after exposure to ASH and mix may be partially due to an anxiolytic effect^49^. Therefore, ASH and Mix show partial anxiolytic effects similar to CLO in OFT. In a previous study, we demonstrated the anxiolytic effect of ASH in the IEBW test^16^. ASH extract administration extended staying time in the open arm and ameliorated the elevation of SNS activity and suppression of PNS activity in the IEBW test. We identified effective constituents by comparing our results with previous findings while investigating the effects of ASH and its components. CHA extended the staying time of rats in the open arm and reduced the suppression of PNS activity in the IEBW test; SYG ameliorated the elevation of SNS activity and the suppression of PNS activity. CHA, a well-known antioxidant and polyphenolic compound, was previously reported to exert anxiolytic effects by enhancing GABA system activity via benzodiazepine receptors^30^. In addition, CHA did not alter time spent in the center area in the OFT^50^. Responses to CHA were consistent with previous findings. Moreover, CHA attenuated suppression of PNS activity induced by stress, a finding consistent with a report that CHA enhances PNS activity in humans^51^. SYG, a lignan glycoside present in ASH roots, is reported to reverse behavioral impairment induced by sleep deprivation by altering hippocampal serotonin and dopamine concentrations^26^. The aglycone of SYG also shows neuromodulating effects^27^. ASH extract, including the aforementioned components, is also reported to influence monoamine levels in rat brain^47^. In the present study, we observed that SYG regulated the cardiac ANS activity (significant) and changed the behavior of rodents (not significant). These effects are believed to be attributable to neuromodulation reported in previous studies. The Mix treatment revealed the anxiolytic effects on autonomic nervous activity and behavior in the IEBW. The modulation of ANS by SYG and Mix is the partial result of anxiolytics for the following reasons. Each treatment affected ANS activity in the home cage. The autonomic modulation against stress generally occurs after stress cognition. Thus, the autonomic modulatory effect by SYG and Mix was considered as the partial result of the anxiolytic effects. Taken together, the results seen in the Mix group rats were consistent with results for whole ASH extract. Therefore, the anxiolytic effects of ASH extract are more likely to be induced through the combined behavioral effects of CHA and neurologic effects of SYG.

We used CLO as a positive control in this study. In general, benzodiazepines, the major class of anxiolytics, increase the time spent in the center area and locomotor activity in the OFT. In the EPM test, benzodiazepines increase entry into and time spent in the open arm. In contrast, CLO only increased locomotor activity in the OFT and had no effect on time spent in the open arm in the IEBW test. The effect of benzodiazepines on the cardiac ANS activity in humans has been reported in many studies^52–59^, but the results are inconsistent. These reports used different conditions, e.g., the differences in duration, use of stressors, use of anesthesia, and underlying diseases. A report by Cloos JM et al.indicates that the effects of benzodiazepines–sedation, muscle relaxation, anticonvulsant effects, and anxiolytic effects–differ among drugs^60^. In their report, CLO was described as follows: sedation, weak; muscle relaxation, weak; anticonvulsant effect, weak; and anxiolytic effect, strong. In our experiments, CLO exhibited beneficial effects in the OFT, associated with mild anxiety, but no effects were observed in the IEBW test, associated with severe anxiety. In contrast, the mixture of ASH constituents (CHA and SYG) was effective for either level of anxiety. During return to closed arm in the CLO group, muscle relaxation was also observed. Further, the number of sleeping rats was higher in this group (data not shown). Therefore, CLO appears to exert anxiolytic effects affecting ANS, but may also be linked to a process that induces muscle relaxation or sleep. Thus, the use of CLO should be closely monitored. This study provides additional information on the limits of anxiolytic effects of CLO on behavior (OFT and IEBW) and ANS activity (IEBW).

Recent studies reported that BDNF–TrkB signaling is important for brain signaling and synaptic plasticity^61^ and is related to various psychiatric disorders, such as depression, bipolar disorder, schizophrenia, panic disorder, and post-traumatic stress disorder (PTSD)^62,63^. Further, previous studies found that hippocampal BDNF expression is also related to anxiolytic effects^35–40^. Hippocampal BDNF mRNA expression is downregulated by both acute and chronic stress^64^, and hippocampal BDNF and TrkB protein expression is also related to vulnerability to stress-induced depression^65^. These findings suggested that anxiety-related behaviors due to stress are closely related to changes in hippocampal BDNF expression. In addition, CREB phosphorylation plays an important role in mediating BDNF-mediated responses in neurons^66^. Anxiolytic effects of ASH under stressful conditions are possibly influenced by changes of hippocampal BDNF expression. Thus, we performed western blotting of BDNF-/TrkB-related proteins in the hippocampus along with immunostaining using a specific anti-BDNF antibody from ASH constituent-administered rat brains after the performance of the IEBW test. Our results showed that BDNF expression and CREB phosphorylation are increased in the hippocampus of SYG-treated rats. Further, administration of Mix increased the protein expression of BDNF and phosphorylation of TrkB and CREB in the rat hippocampus. Both ASH and the aglycone of SYG influence levels of hippocampal monoamines and metabolites^27,47^. Noradrenaline, dopamine, and serotonin are related to hippocampal BDNF expression^67–69^. Therefore, these monoamines are positively related to BDNF expression.

Immunohistochemical analysis indicated that BDNF expression in rat hippocampus is markedly elevated under IEBW conditions following treatment with SYG and Mix. Expression changes in CA1 and partial DG regions differed between the SYG and Mix group animals. The CA1, CA3, and DG regions of the hippocampus are vulnerable to glucocorticoid toxicity^70^, and corticosterone, the major glucocorticoid in rodents, and stress both reduce hippocampal BDNF expression^64,71–73^. Corticosterone levels are higher in pyramidal neurons in CA1 and granule neurons in DG than that in other areas of the hippocampus^74^. Glucocorticoid receptor is abundantly expressed in CA1 and DG, but expression is lower in CA3. Thus, the influence of corticosterone on stress in the hippocampus differs by region. In particular, the volume of CA1 is smaller in patients with PTSD, and chronic downregulation of BDNF mRNA expression in this area leads to PTSD-like behavioral stress responses. A smaller CA1 volume has also been reported in Alzheimer’s disease and schizophrenia^75,76^. Xiao yao san, a well-known traditional Chinese medicine formula, has anti-depressive effects and counteracts the reduction of BDNF expression in CA1 induced by chronic immobilization stress^77^. Further, Jessica CJ et al.reported that the cells responsible for defensive behavior against anxiogenic environments are located in the ventral CA1 region^78^. BDNF in the CA1 region might modulate anxiety cells. Therefore, Mix, which increased BDNF protein expression in the CA1 region, a finding not observed in the SYG group, could be useful for treating PTSD, Alzheimer’s disease, and schizophrenia.

Our findings show that CHA and SYG deferentially affect ANS activity and behavior. In addition, the combination of these constituents plays important roles in the anxiolytic effects of ASH extract and its ability to activate hippocampal BDNF signaling. Taken together, our findings identified CHA and SYG as the effective constituents of ASH extract, suggesting that they could be used in combination as a beneficial supplement or preventive medicine for the maintenance of mental health.

## Materials and methods

### Materials

#### Plant extract

A dried 2–5-cm root tip of ASH from Heilongjiang, China was cut and extracted using hot water. The extract was concentrated under reduced pressure and dried using a spray dryer. The extract powder (Lot No. 8142) was provided by Sun Chlorella Co., Ltd. (Kyoto, Japan). We previously measured each major component in the extract using HPLC and an ODS column^16^. ASH extracts are primarily composed of isofraxidine (101.4 mg/100 g), eleutheroside B (325.2 mg/100 g), ( +)-syringaresinol–di–O–β-D-glucoside (625.2 mg/100 g), eleutheroside B1 (95.2 mg/100 g), and CHA (829.5 mg/100 g).

#### Isolation of SYG

The extract powder of ASH (1250 g) was extracted using methanol (3750 mL, 3 h × 2) under a reflux condition. The extract was filtered, followed by concentration to a small volume under reduced pressure. The given syrup was suspended in H_2_O (volume) and extracted successively with Et_2_O, CHCl_3_, and *n*-BuOH. The *n*-BuOH extract (219 g) was separated into five portions, and each portion (approximately 41 g) was subjected to SiO_2_ column chromatography (Ø 8.0 × 25.0 cm) and eluted with CHCl_3_/MeOH/H_2_O (450:50:5). The fractions containing the compound with an *Rf* value of 0.55 (CHCl_3_/MeOH/H_2_O = 70:30:5) were collected and concentrated. The given residue was further purified via SiO_2_ column chromatography (Ø 3.0 × 19.0 cm) and eluted with EtOAc/CHCl_3_/MeOH (2:2:1). The fractions containing the compound with an *Rf* value of 0.15 (EtOAc/MeOH = 7:3) were collected and concentrated. Recrystallization from MeOH gave 650 mg of white precipitate. The purity was measured using the UV absorption at 220 nm on a COSMOSIL 5C_18_-AR-II column (4.6 × 250 nm, NACALAI TESQUE, INC., Kyoto, Japan). The solvents for HPLC were as follows: A, 0.05% TFA in water; and B, 0.05% TFA in acetonitrile. The detection column was eluted at a flow rate of 1 mL/min with a linear gradient of 90% for 5 min followed by 90% A to 50% A over 40 min. The retention time (*t*_R_) is reported in minutes (*t*_R_ = 22.2 min, 91.0% purity). The isolated substance (ESI–MS) had the molecular formula C_34_H_46_O_18_ (742.2684 g/mol) and m/z [M + Na]^+^ 765.2618 (calcd, 765.2582).

### Animals

Male Sprague Dawley rats (6 weeks old in the IEBW test, 7 weeks old in the OFT) were purchased from SLC, Inc. (Shizuoka, Japan), individually housed in standard polycarbonate cages for 7 days and subjected to serial 7-day handling. Normal diet (ND, MF, Oriental Yeast Manufacturing Co., Ltd., Tokyo, Japan) and water were available ad libitum. Rats were kept in a room maintained at 23 °C ± 2 °C. After 1-week acclimatization, rats were implanted the telemeter in the abdomen (see Supplementary Materials and Methods 1.1 for details). Based on the test food consumption in our previous study of the ASH extract^16^, we determined the dose of each test item (CHA[Sigma-Aldrich, C3878], 40 mg/kg; SYG, 32 mg/kg; Mix, a mixture of CHA and SYG) dissolved in distilled water. CLO (SEPAZON, Alfresa Pharma Co., Osaka, Japan) was administered at a dose of 0.2 mg/kg in 1 mL of distilled water. All test items were administered through a probe once a day for 7 days. On the behavioral test day, rats were administered 1 mL of each solution 0.5 (Cont, CHA, SYG, and Mix) or 2 h (CLO) before each behavioral test. In the OFT, we administered ASH as per the previous study^16^.

### Behavioral test

#### OFT

**Figure 6:**
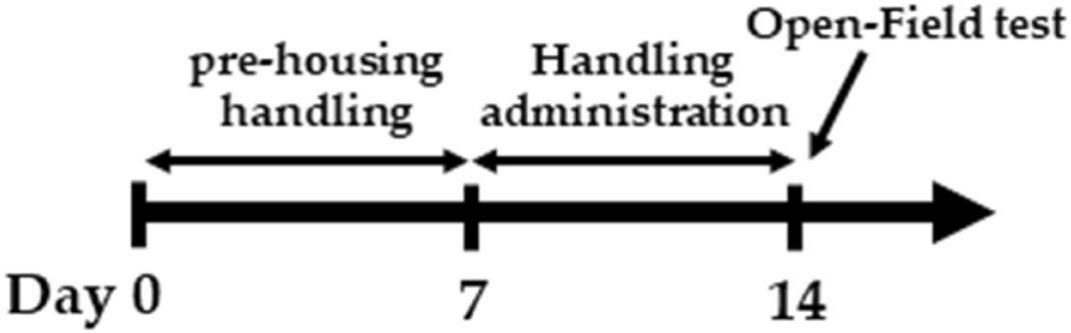
Schedule of the open field test (OFT). After 1 week of pre-housing, rats were administered 1 mL of each reagent. On the test day, rats were administered the test agents 0.5 or 2 h before the OFT. Rats were then placed in the apparatus and allowed to explore the area freely for 10 min.

The OFT is often used as a measure of anxiety-like behaviors, and it was conducted as described by Broadhurst^79^ with modifications. The OFT schedule is presented in Fig. 6. We used a commercial open field apparatus (OF-25R, 75 cm diameter with 40 cm height wall, Muromachi Kikai, Tokyo, Japan). The apparatus was divided into three sections as described in Fig. 1C. Rats were placed into the open field apparatus and allowed to explore the area freely for 10 min. The activity in the open field apparatus was recorded by a video camera, and activity was analyzed using ANY-maze software (Stoelting Co., Wood Dale, IL, USA) We assessed the total distance traveled, total time mobile, total entries into each area, and the ratio of entries into each area to the total entries.

#### IEBW test

**Figure 7:**
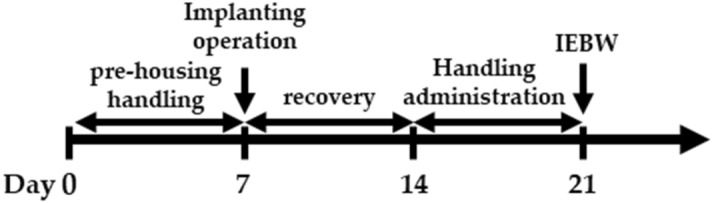
Schedule of the improved elevated beam walking (IEBW) test. After 1 week of pre-housing, the rats were implanted with a telemeter followed by 1 week of recovery. After the recovery period, the rats were administered 1 mL of each reagent. On the test day, rats were administered reagents 0.5 or 2 h before the IEBW test. Rats were placed in the apparatus and allowed to explore the area freely for 3 min.

We conducted behavioral experiments in rats subjected to high-fear stress to examine anxiolytic effect of ASH components. In general, the EPM test is conducted to evaluate anxiety in animals^80^. We used the IEBW apparatus by installing a 2 × 4 timber (180 × 8.9 cm) 190 cm above the floor level. The apparatus included open (140 × 8.9 cm) and closed (40 × 8.9 × 28.5 cm) arms as described previously^16^. The IEBW test schedule is described in Fig. 7. After seven days of treatment, rats were placed in the tip of the open arm 140 cm away from the closed arm, and their behavior and HRV examined for 3 min (IEBW test). We then assessed the time spent in the open arm, LF/HF, LFnu, and HFnu.

### Brain tissue preparation

30 minutes after behavioral tests, rats were euthanized, and their brains were rapidly excised. Hippocampal tissues were stored at − 70 °C until western blotting.

### Assessment of cardiac ANS activity (HRV)

After the operation (see supplementary materials), ECG data were continuously recorded using the radio-telemetry system, comprising the implanted device, a battery-charging and telemetry-receiving device underneath the cage, and a data acquisition system (PowerLab16/35, PL3516, AD Instruments, Castle Hill, New South Wales, Australia) interfaced with a computer. Cardiac autonomic activity was assessed via spectral analysis of R-R interval variability. Data were stored and analyzed using LabChart Pro (ver. 8.0, AD Instruments). Frequency domain analysis and power spectra of R-R interval variability were obtained using the fast Fourier transform algorithm. High (HF: 0.6–3.0 Hz), low (LF: 0.2–0.6 Hz), and very low (≤ 0.2 Hz) frequencies were determined. LF and HF components were expressed in normalized units (LFnu and HFnu). The power of the HF component indicates cardiac parasympathetic activity, whereas that of the LF component indicates the sympathetic activity with parasympathetic modulation. LF/HF is an index of the cardiac sympathetic-parasympathetic tone balance.

### Western blotting

Western Blotting for analysis hippocampal protein expression and phosphorylation was conducted as our previous report^16^. Homogenized hippocampal lysates were separated by 10% sodium dodecyl sulfate–polyacrylamide gel electrophoresis and transferred to Amersham Hybond P PVDF 0.45 membranes (Bio-Rad, Hercules, CA, USACytiva, Tokyo, Japan). Membranes were incubated with 5% bovine serum albumin (BSA) in TBST for 1 h, followed by incubation with primary antibodies against BDNF (ab108319), TrkB; #4603S), pTrkB (#4619S), pCREB (#9198S), CREB (#9197S), and β-actin (#4970S) at a 1:1000 dilution overnight at 4 °C. Anti-BDNF antibody was bought from Abcam (Cambridge, UK), and other primary antibodies were purchased from Cell Signaling Technology Japan, K.K. (Tokyo, Japan). This was followed by incubation with horseradish peroxidase-conjugated secondary antibodies (#7074S, 1:1000, Cell Signaling Technology Japan) for 1 h. Immunoreactive bands were detected using an enhanced chemiluminescence detection kit, and a Light Capture AE-6971/2 device (ATTO Corp., Tokyo, Japan) was used for visualization. Band intensities were normalized to β-actin, total TrkB, or CREB using CS Analyzer 4 (ATTO Corp.).

### Immunohistochemistry

Immunohistochemistry for analysis hippocampal BDNF expression was conducted as previous our report^16^. Rat brains were fixed overnight in 4% paraformaldehyde, followed by immersion in graded sucrose solutions for cryopreservation (10, 20, and 30% sucrose in phosphate-buffered saline [PBS]) and freezing on dry ice. Frozen brains were mounted using Tissue-Tek O.C.T. compound (Sakura Finetek Japan Co., Ltd., Tokyo, Japan) and stored at − 80 °C. Brains were then sectioned into 10-µm-thick sections using a cryostat at − 15 °C. Sections were collected in PBS and incubated with 10% BSA in PBS for 1 h, followed by incubation with anti-BDNF antibody (sc-20981, 1:100, Santa Cruz Biotechnology, Santa Cruz, CA, USA) for 3 days at 4 °C. Incubation with the secondary antibody (#4413S, 1:1000, Cell Signaling Technology Japan) proceeded for 2 h, after which sections were mounted onto MAS-coated glass slides and cover-slipped using Fluoromount (Diagnostic BioSystems, Pleasanton, CA, USA). Images were obtained using a fluorescence microscope (BZ-9000, Keyence, Osaka, Japan).

### Statistics analysis

Values were expressed as the mean ± standard error and derived from measurements of 5–7 rats (OFT, n = 5–6; IEBW test, n = 5–7, western blotting, n = 5). Statistical analysis was performed using SPSS statistics 25 (IBM Japan, Ltd., Tokyo, Japan). Homogeneity of variances was checked using Levene’s test. One-way ANOVA was used for inter-group comparisons. When ANOVA revealed significant differences, Dunnett’s *t*-test or T3 post hoc test was used to identify significant differences versus the Cont group. Differences between two groups were analyzed using a paired Student’s *t*-test. *p* < 0.05 denoted statistical significance.

### Ethics statement

This study was conducted according to the “Guide for the Care and Use of Laboratory Animals” (NIH Publication No. 85–23, revised in 1996). All experimental protocols were approved by the Ethics Committee on Animal Use of Suzuka University of Medical Science (No. 1 of April 1, 2016).

## Supplementary information

Supplementary Information 1.

Supplementary Information 2.
