# Supplementary material for: Combination of syringaresinol–di–O–β-d-glucoside and chlorogenic acid shows behavioral pharmacological anxiolytic activity and activation of hippocampal BDNF–TrkB signaling
Source: Sci Rep. 2020 Oct 23;10:18177. doi: 10.1038/s41598-020-74866-4 (PMC7584579; doi:10.1038/s41598-020-74866-4)

**Combination of syringaresinol–di–O–β-d-glucoside and chlorogenic acid shows behavioral pharmacological anxiolytic activity and activation of hippocampal BDNF–TrkB signaling**

**Shouhei Miyazaki, Yoshio Fujita, Hirotaka Oikawa, Hideo Takekoshi, Hideaki Soya, Masato Ogata and Takahiko Fujikawa^*^**

**1. Supplementary Materials and Methods**

*1.1 Surgery*

After 1-week acclimatization, rats were treated with a local anesthetic (ropivacaine hydrochloride hydrate) and general anesthesia (sodium pentobarbital, 40 mg/kg, i.p.), and a wireless telemeter (model TR50BB; KAHA sciences Ltd., Auckland, New Zealand) was implanted in the abdomen. Animals were then intramuscularly injected with antibiotics (imipenem hydrate and cilastatin sodium, 8.3 mg/kg) to prevent postoperative infections. After the operation, rats were allowed to recover for 7 days.

*1.2 Animal selection*

To reduce the variability of the data and increase the reproducibility of the experiment with minimum sample size, we used the following original selection method. Concerning behavior in the home cage, we consider it necessary for the rat to be at the near side of the home cage when an experimenter is present. If the rats become aware that the experimenter may be dangerous, they will be wary and move to the far side of the home cage. If the experiment was repeated, rats on the far side of the home cage sometimes showed strong avoidance behaviors (e.g., they became violent and tried to escape from the cage during handling) or showed depressive behavior. In contrast, rats on the near side of the home cage did not show such behaviors. Hence, we recorded the position of the rats in the home cage both after entering the breeding facility and after handling and used only the rats on the near side of the home cage. In addition, we examined only those rats in which ANS was not disturbed by the handling of the experimenter for reducing the variability. Such rats showed less disruption of autonomic activity during handling and showed higher reproducibility in behavioral pharmacology tests.

*1.3 Details of improved elevated beam walking (IEBW) test*

In the IEBW test, we recorded rats visually because of the apparatus height, but we excluded rats that did not move from the tip of the open arm or with extremely low locomotor activity from the analysis. We placed the rats in the closed arm to explore only the closed arm for 3 minutes for 2 consecutive days before initiating the treatment and selected rats that could enter the closed arm from 10, 20, or 50 cm distance for the experiment. In this experiment, the rats moved 120 cm to enter a closed area, and the time spent on IEBW was measured.

**2. Supplementary Figure**

**Supplementary Figure 1** Western blotting; full length blots of (A) β-actin, (B) BDNF, (C) pTrkB, (D) TrkB, (E) pCREB, and (F) CREB. The number above the lane represent each groups 1: Cont, 2: CHA, 3: SYG, 4: Mix. The part surrounded by dashed lines is shown in the manuscript.


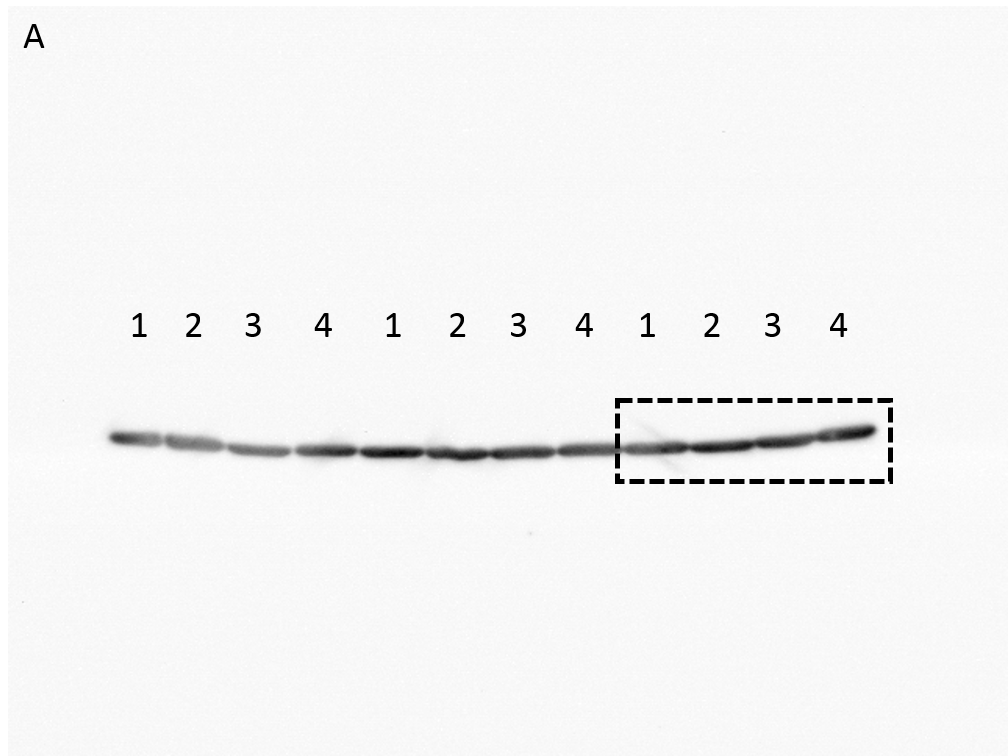

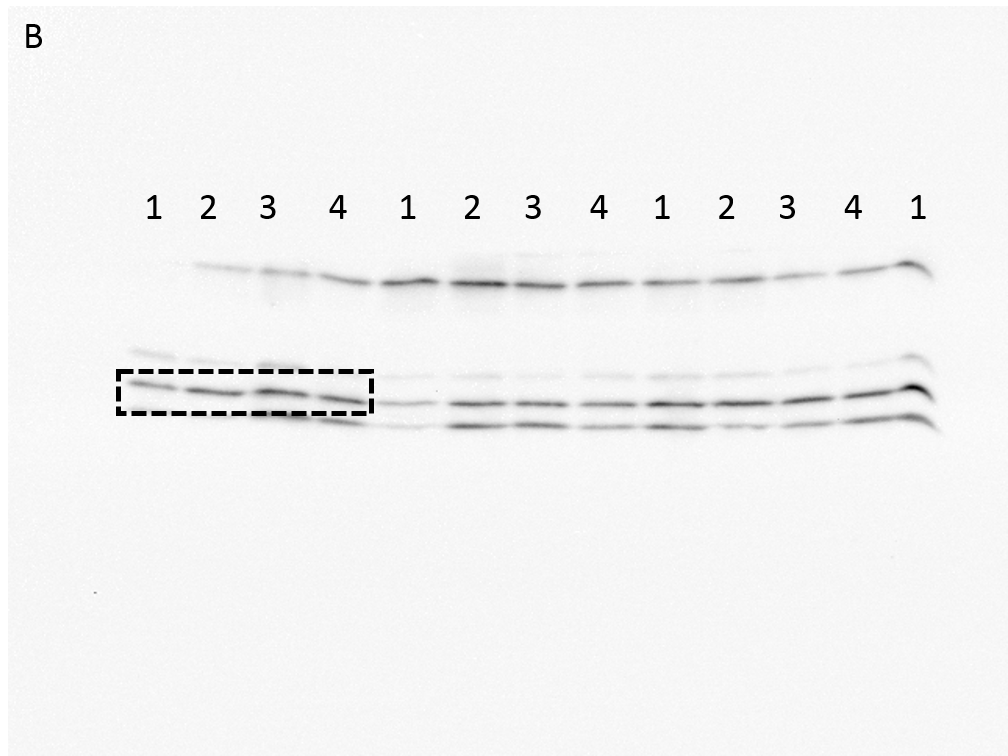

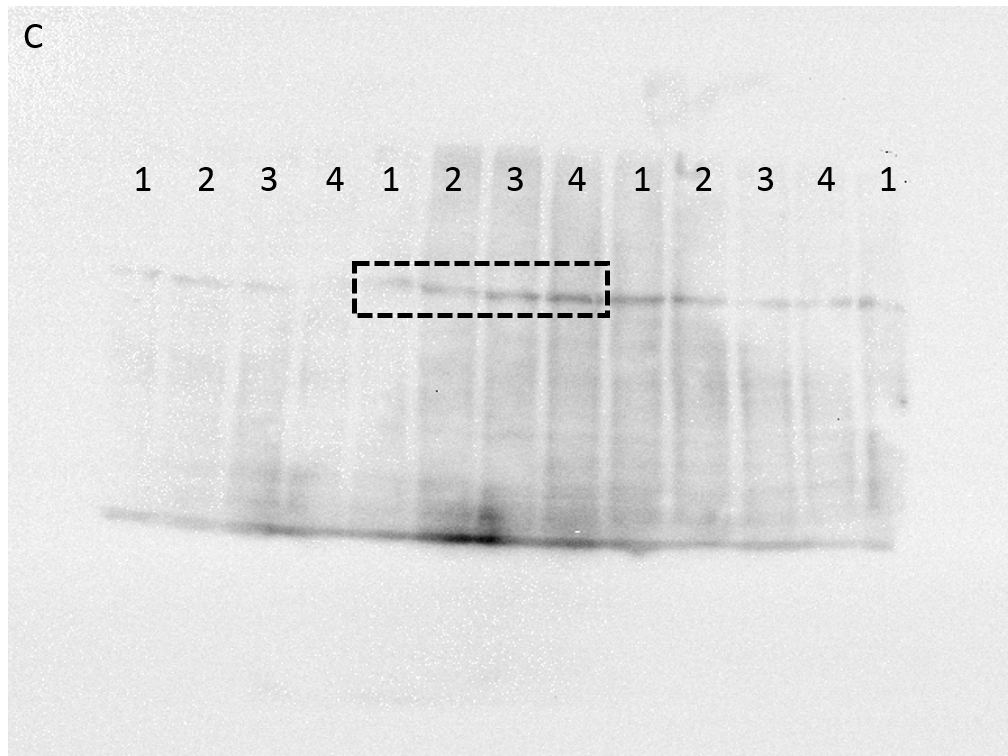

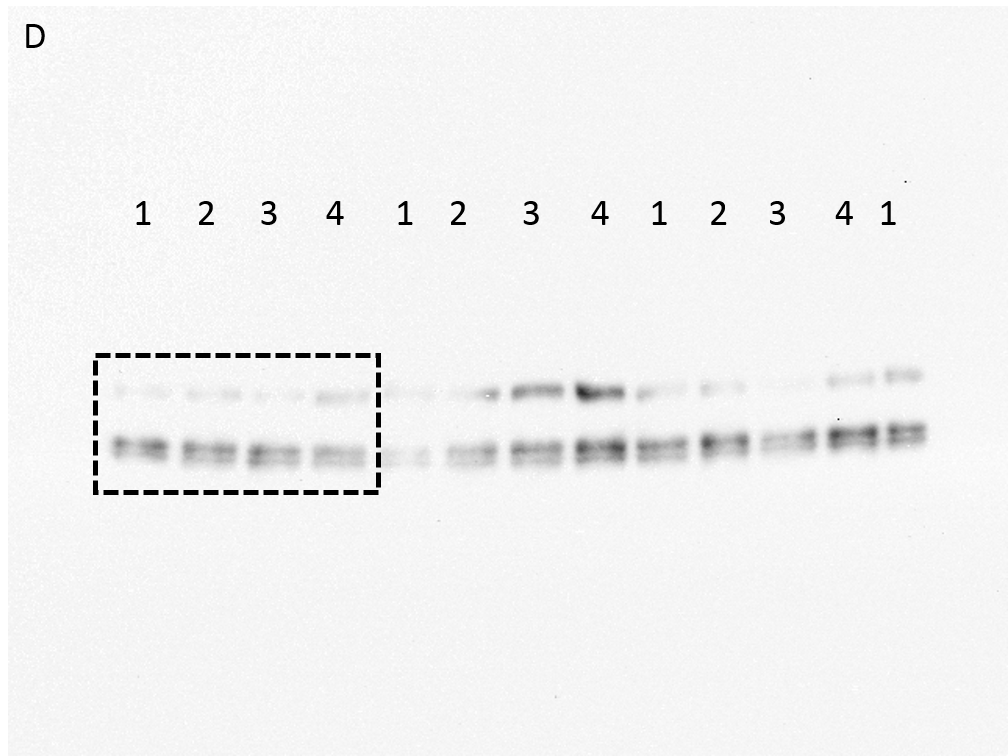

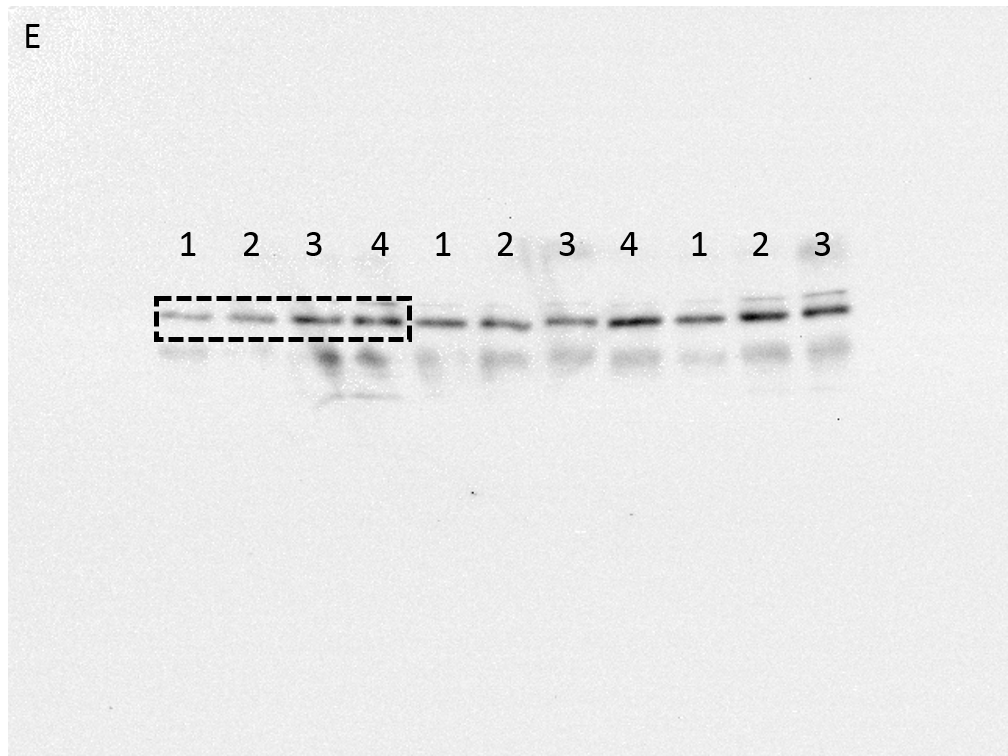

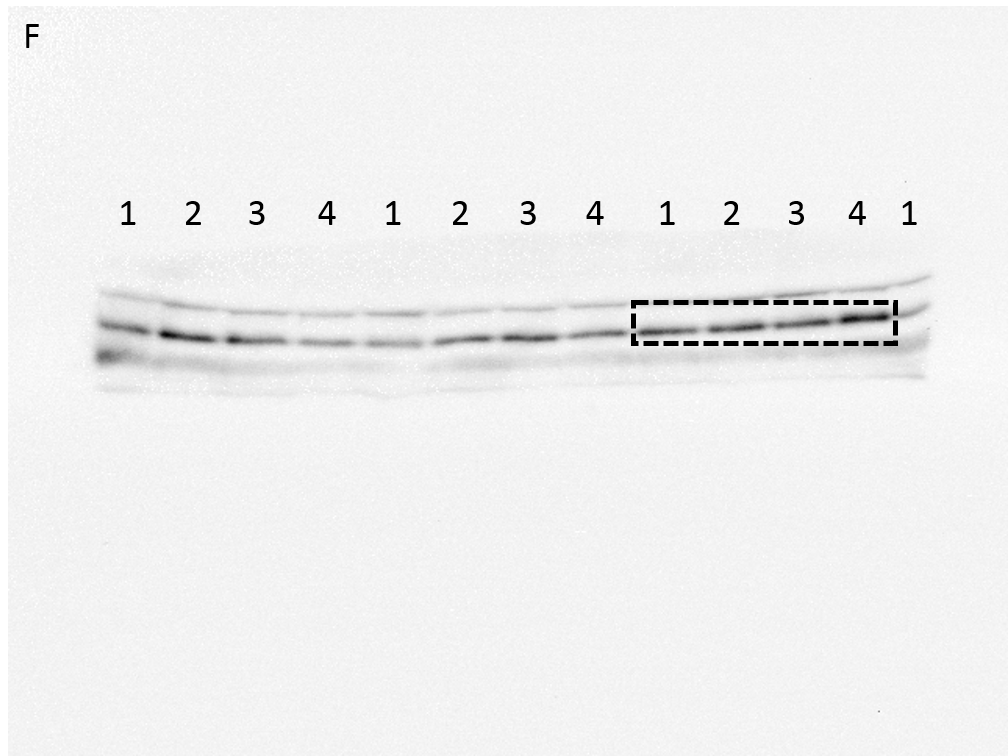

Supplement: Supplementary file 1 — Supplementary Information 1. [file 41598_2020_74866_MOESM1_ESM.docx]
